# Incidence of palliative treatment among breast cancer patients undergoing neoadjuvant therapy: an analysis of the Brazilian public health system

**DOI:** 10.1038/s41598-025-06113-7

**Published:** 2025-07-01

**Authors:** Ana Maria Fantini Silva, Breno Piva, Marco Antônio Prado Nunes

**Affiliations:** 1 Health Sciences Graduate Program (PPGCS), Federal University of Sergipe, Aracaju, Sergipe Brazil; 2 Department of Medicine (DME), Federal University of Sergipe, Campus Prof. João Cardoso Nascimento, Aracaju, Sergipe Brazil; 3 Computing Department (DCOMP), Federal University of Sergipe, Cidade Universitária Prof. José Aloísio de Campos, São Cristóvão, Sergipe Brazil

**Keywords:** Cancer, Oncology

## Abstract

Breast cancer is the leading cause of cancer-related deaths among women globally, including Brazil. This study assesses neoadjuvant treatment patterns, the impact of new therapies, and factors determining palliative care for patients who failed initial treatments. A historical cohort study using secondary data from DATASUS (2008–2017) focused on women aged 18–90 with stages II and III breast cancer who received neoadjuvant treatment. Data on chemotherapy, hormonal therapy, and anti-HER2 treatment were analyzed. The Palliative Treatment Rate (PTR) was calculated by cross-referencing neoadjuvant treatment records with subsequent palliative care records, indicating cases where the initial curative intent was not achieved. The study included 71,181 patients, with a mean age of 51.5 years. Most were diagnosed at Stage III (85%). Anti-HER2 therapy was introduced in 2013, with 10.5% receiving it. The 5-year PTR decreased from 44% (2008–2012) to 36% (2013–2017). The combination of chemotherapy, endocrine therapy, and anti-HER2 therapy had the lowest PTR. Logistic regression identified younger age, higher histological grade, Stage III disease, and compromised lymph nodes as factors increasing the likelihood of needing palliative care, while HER2 positivity and hormonal therapy reduced it. Regional disparities were observed, with patients from the Southeast more likely to receive palliative care. Early diagnosis and access to anti-HER2 therapy significantly reduce palliative care needs. Socio-economic and regional disparities in treatment highlight the need for equitable access to diagnostic tools and therapies to improve survival outcomes in Brazil’s public healthcare system.

## Introduction

Breast cancer represents the leading cause of cancer-related mortality among women in Brazil and globally. Excluding non-melanoma skin cancers, it is the most prevalent malignant neoplasm in the female population^1–3^.

According to the World Health Organization, based on estimates for the year 2020, in countries with a very high Human Development Index (HDI), the lifetime risk of a woman being diagnosed with breast cancer is approximately 1 in 12, with a mortality risk of 1 in 71. In contrast, in countries with a low HDI, the lifetime diagnosis rate is around 1 in 27, and the mortality rate is 1 in 48^4^.

In developing nations like South Africa and Brazil, insufficient funding and the lack of clearly defined preventive measures lead to reduced survival rates and delayed diagnoses. Consequently, a larger percentage of patients are diagnosed with locally advanced breast cancer^4–7^.

In Brazil, approximately 75% of the population depends exclusively on public healthcare services. Barriers to accessing high-complexity care for initiating cancer treatment — such as geographic limitations, lack of available appointments, surgical delays, or unavailability of oncologic therapies — can negatively affect patient outcomes, compromising both cure rates and overall survival^6,8^. Data from a recent trial showed that among patients with locally advanced disease (Stage III), the 5-year overall survival rate was 65%, and disease-free survival was 47% for those treated in public services, whereas in private services, these rates were 95% and 61%, respectively^9^.

Breast cancer incidence and mortality vary significantly across regions in Brazil. Although the North has a smaller population and lower overall incidence rates, it presents a disproportionately high mortality in relation to the number of diagnosed cases. This suggests a greater risk of death from breast cancer in that region, likely reflecting limitations in access to early diagnosis and adequate treatment, as well as structural disparities within the healthcare system^1,10^.

Locally advanced breast cancer necessitates complex multidisciplinary treatment, including neoadjuvant chemotherapy, surgery, and radiotherapy. Advances in understanding tumor biology and the development of new oncological treatments have substantially improved disease control and patient survival under controlled conditions^11^.

The aim of this study was to assess the landscape of neoadjuvant breast cancer treatment in Brazil’s Public Health System, evaluate the impact of the incorporation of new medications, and outline the determining factors for the subsequent administration of palliative treatment in patients who failed the initial curative approach.

## Method

### Study design

This is a secondary data study, based on the construction of a historical cohort using a Data Warehouse created through the linkage of information obtained from official government databases. Publicly available data from DATASUS, which is responsible for chemotherapy and radiotherapy procedure data, were utilized. This study is part of a series of studies on neoadjuvant breast cancer treatment within the Brazilian public health system.

### Study population

The study included exclusively data on patients who received neoadjuvant treatments initiated between January 2008 and December 2017, involving female patients aged 18 to 90 years old diagnosed with initial stages II and III breast cancer. According to the Brazilian public health protocol in effect until 2024, neoadjuvant therapy for breast cancer was limited to patients with stage III disease. However, a preliminary assessment revealed a non-negligible number of stage II patients undergoing neoadjuvant treatment in clinical practice, which justified their inclusion in the present study.

Patients whose interval between cancer diagnosis and the initiation of neoadjuvant treatment was negative—due to data entry errors, since the diagnosis date must precede the start of oncologic treatment—or exceeded 180 days were excluded. The 180-day limit was based on similar studies evaluating delays in treatment initiation^12,13^. The estimated proportion of exclusions due to intervals longer than 180 days was approximately 5% of the study population.

### Data collection and variables

A script was developed in the R programming language for the extraction, transformation, and loading (ETL) of data into the Data Warehouse, using information provided by official databases.

To identify cases in which there was a failure of the initial curative approach, any treatment—chemotherapy, hormone therapy, or radiotherapy—coded in the DATASUS database as having palliative intent was considered as palliative. The Palliative Treatment Rate (PTR) was calculated by cross-referencing the date of diagnosis, as recorded in the neoadjuvant treatment authorization, with the first palliative treatment recorded in the system within five years. This method allowed the identification of patients who progressed or relapsed after neoadjuvant therapy and subsequently required palliative management.

Sociodemographic information, such as age, residential municipality, and treatment municipality, as well as oncological data, including histological grade, presence of compromised lymph nodes at the start of treatment, and staging, were analyzed to identify factors associated with a higher frequency of palliative treatment.

For tumor-related and sociodemographic variables, missing or unclassifiable data were grouped under the category ‘ignored/not informed. It was not possible to evaluate whether patients underwent surgery after neoadjuvant chemotherapy, as surgical procedure records could not be reliably cross-referenced with the chemotherapy and radiotherapy databases.

### Statistical analysis

Statistical analysis was performed using a range of methods, including descriptive statistics and hypothesis testing. Descriptive statistics such as mean, median, standard deviation, interquartile range, frequency counts, and percentages were used to characterize the variables and summarize the collected data. The Chi-square test was employed to assess the association between categorical variables, determining whether observed frequencies differed from expected frequencies, which may indicate statistically significant associations. For analyses involving palliative treatment, an expected palliative treatment rate of approximately 30% was considered, based on prior epidemiological studies^9,33^, to contextualize the observed findings.

To evaluate prognostic factors, logistic regression was utilized. This method models the probability of a binary event—such as whether or not palliative treatment was administered—and transforms predictions into probabilities between 0 and 1 using the logistic function. Logistic regression coefficients are interpreted as odds ratios, indicating the change in the odds of the event with a one-unit change in the independent variable. Poisson regression was applied for count data, where the dependent variable represents the number of occurrences of an event within a given time or space interval, assuming a Poisson distribution^14^.

Kaplan-Meier estimation was used to analyze survival times, allowing for the visualization of survival curves for different treatment groups. The Log-rank test was applied to test the hypothesis that the survival curves of the groups were equivalent^15^. The follow-up period for this analysis spanned from 2013 to 2022.

All statistical analyses were conducted using R software (version 4.3.2) (R CORE TEAM, 2023), with a significance level set at 5%^16^.

## Results

### Baseline characteristics

A total of 71,181 patients were included in the study. The mean age was 51.54 years, ranging from 19 to 90 years. Table 1 shows that 44.4% of neoadjuvant treatments were administered to patients from the Southeast region of Brazil, while only 3.37% of women receiving neoadjuvant treatment resided in the North region. There was a predominance of white women, followed by mixed-race and black women.

Regarding neoplasm characteristics, the majority of patients had compromised axillary lymph nodes at the beginning of treatment, and there was a predominance of Grade II neoplasms. Approximately 87% of women were diagnosed at stage III (Table 1).

**Table 1:** Study participants demographic and clinical-pathological characteristics.

Characteristic	*N* (71,181)	%
Age (Years)		
≤ 40	13,595	19.10%
40–50	21,581	30.32%
50–60	19,217	27.00%
60–70	11,656	16.38%
> 70 anos	5,132	7.20%
Patient Race / Ethnicity		
No information	14,670	20.61%
White	31,519	44.28%
Black	3,659	5.14%
Mixed races	20,016	28.12%
Asian	1,303	1.83%
Indigenous	14	0.02%
Regional Lymph Node Involviment		
Yes	37,521	52.71%
No	16,751	23.53%
Not Assessed	16,909	23.76%
Tumor Grade		
Grade I	4,610	6.48%
Grade II	21,534	30.25%
Grade III	17,075	23.99%
Grade IV	867	1.22%
Indeterminate	27,095	38.06%
Clinical Stage		
Stage 2	9,618	13.51%
Stage 3	61,563	86.49%
Brazilian Region		
Northeast	19,639	27.59%
Midwest	4,143	5.82%
North	2,527	3.55%
Southeast	31,320	44.44%
South	13,240	18.60%

N: number of patients; %: percentage of the total sample.

### Treatment

Table 2 describes neoadjuvant treatments. About 97.5% of patients commenced treatment with neoadjuvant chemotherapy, with 4.4% (3,136 patients) having their regimen changed to chemotherapy plus anti-HER2 therapy during the neoadjuvant phase. Fewer than 2.0% received chemotherapy combined with anti-HER2 therapy from the start of neoadjuvant treatment, and only 0.5% were treated with hormonal therapy during the neoadjuvant phase. The first use of anti-HER2 therapy code in neoadjuvant treatment occurred in February 2013.

Considering only the period from 2013, when anti-HER2 therapy was officially incorporated into the Brazilian public health system, 4,416 patients (10.5%) received neoadjuvant anti-HER2 therapy, with 1,326 patients (30.03%) receiving it from the beginning of treatment.

**Table 2:** Neoadjuvant treatments performed between 2008 and 2017.

Neoadjuvant	*N*	%
**2008–2017**	**71,181**	
Prior Chemotherapy	66,261	93.08%
Prior Chemotherapy -> anti-HER2 therapy	3,136	4.40%
anti-HER2 therapy	1,385	1.94%
Endocrine Therapy	399	0.56%
**2008–2012**	**28**,**316**	
Prior Chemotherapy	28,211	99.64%
Prior Chemotherapy -> anti-HER2 therapy	96	0.33%
anti-HER2 therapy	9	0.03%
Endocrine Therapy	0	0%
**2013–2017**	**42**,**865**	
Prior Chemotherapy	38,050	88.78%
Prior Chemotherapy -> anti-HER2 therapy	3,040	7.08%
anti-HER2 therapy	1,376	3.21%
Endocrine Therapy	399	0.92%

Regarding adjuvant treatment administered following neoadjuvant therapy, 45.87% (32,651 patients) received hormonal therapy, and 9.49% (6,756 patients) received anti-HER2 therapy in the adjuvant setting over the entire cohort period. Approximately 53% of those who received adjuvant anti-HER2 therapy also received adjuvant hormonal therapy. When considering only the period after the incorporation of anti-HER2 therapy into SUS from 2013 onwards, 14% of patients received adjuvant anti-HER2 therapy (Table 3).

For 17,785 patients (approximately 25% of the sample), the only curative treatment recorded in the Data Warehouse was neoadjuvant treatment, without subsequent radiotherapy and/or hormonal therapy and/or adjuvant anti-HER2 therapy (Table 3).

Regarding radiotherapy, 44,020 patients underwent supplementary radiotherapy, either adjuvant or neoadjuvant, during the study period, corresponding to 61.84% of the population.

**Table 3:** Adjuvant treatments performed between 2008 and 2017.

Adjuvant Therapy	*N* (71,181)	%
Endocrine Therapy	32,651	45.87%
Anti HER2 Therapy	6,756	9.49%
Endocrine Therapy + Anti HER2 Therapy	3,621	5.08%
Radiotherapy	44,020	61.84%
No adjuvante Therapy	17,785	24.98%

At least one type of palliative treatment was administered in 27,991 patients, with 92% occurring within the first 5 years after diagnosis. The 5-year palliative treatment rate was 36.2%. Of the 2,167 patients who started palliative treatment after 5 years from diagnosis, approximately 83% received adjuvant hormonal therapy.

The proportion of patients receiving any palliative treatment decreased from 44 to 36%, with *p* < 0.001 when comparing the periods 2008–2012 and 2013–2017.

**Fig. 1:**
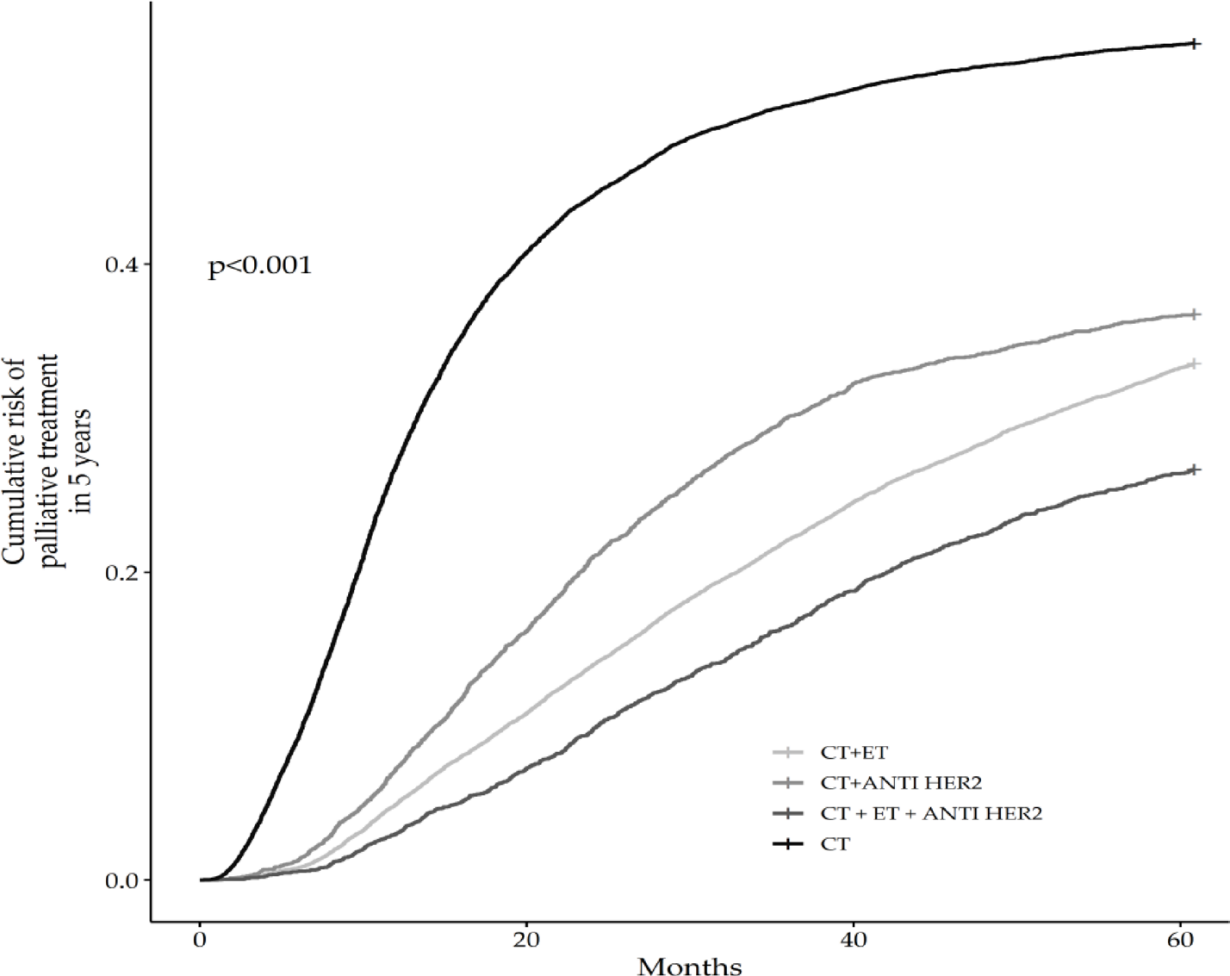
Cumulative risk of palliative treatment in 5 years.

The analysis of the 5-year palliative treatment rate curves for different therapeutic groups shows statistically significant differences in survival between them (*p* < 0.001). Observing the curve, Chemotherapy (CT) + Endocrine therapy (ET) + anti-Her2 therapy group exhibits the lowest palliative treatment rate over time, followed by the CT + ET and CT + anti-Her2 groups. The isolated QT group has the highest palliative treatment rate, as indicated by the Fig. 1.

**Fig. 2:**
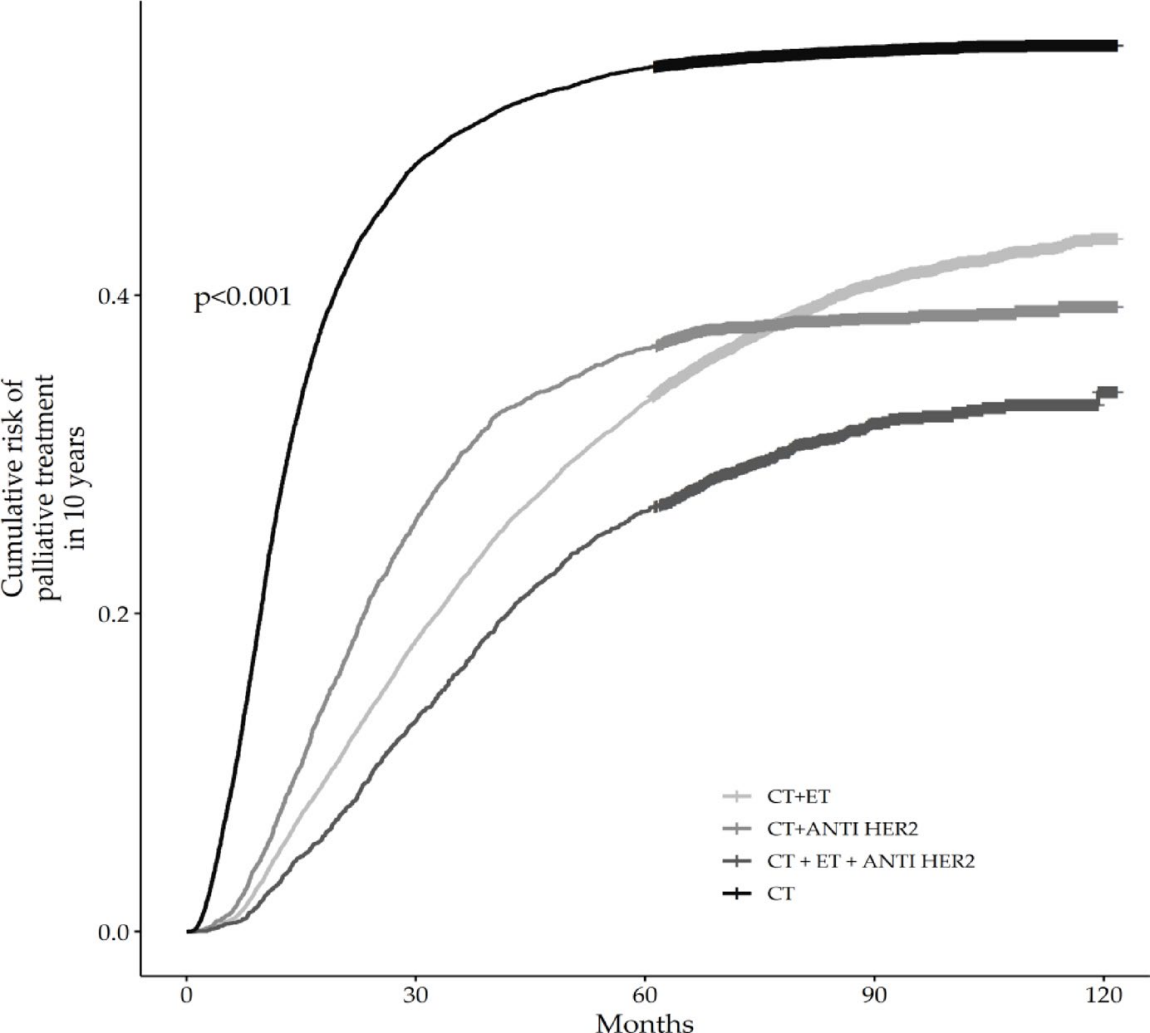
Cumulative risk of palliative treatment in 10 years.

When evaluated over a 10-year period, the paired comparison analysis between treatment groups after 10 years, using the Log-Rank test, reveals statistically significant differences in progression-free survival without palliative treatment. The comparison between CT + anti-Her2 and CT + ET did not show a significant difference (*p* = 1), suggesting that after 10 years, the palliative treatment risk between these two groups are equivalent. On the other hand, comparisons involving the CT + ET + anti-Her2 therapy group with the CT + ET and CT + anti-Her2 groups were significant, with p-values of 1.3e-13 and 4.6e-10, respectively. This indicates that the combination of ET with anti-Her2 in a chemotherapy regimen continues to provide significant advantages in terms of the occurrence of palliative treatments compared to other combinations (Fig. 2). The statistical differences remained when analyzed according to the initial stage.

### Prognostic factors

Table 4 presents the results from the logistic regression model, highlighting several variables with significant associations with the palliative treatment outcome. Patient age at the start of treatment significantly reduced the likelihood of receiving palliative care, with an odds ratio of 0.992 and a p-value < 0.001, indicating that younger patients are more likely to receive palliative treatment.

Histopathological grade also showed significant associations, with Grade I being associated with a decreased likelihood (OR = 0.897, *p* = 0.001) and Grade IV being significantly associated with an increased likelihood of requiring palliative treatment (OR = 1.336, *p* < 0.001). No statistical difference was observed for Grade III. Disease stage had a significant impact, with Stage III associated with an increased likelihood of palliative treatment (OR = 1.317, *p* < 0.001).

The presence of invaded regional lymph nodes increased the likelihood of palliative treatment (OR = 1.089, *p* < 0.001). The presence of HER2 positivity and the use of hormonal therapy were significantly associated with a decreased likelihood of receiving palliative treatment, with odds ratios of 0.674 (*p* < 0.001) and 0.758 (*p* < 0.001), respectively.

Regional differences were also observed, with patients from the North having a lower likelihood of receiving palliative care (OR = 0.791, *p* < 0.001), while those from the Southeast had a higher likelihood (OR = 1.122, *p* < 0.001). Other variables, such as patient race/ethnicity and the use of radiotherapy, did not show statistical significance at the established level (*p* < 0.05).

**Table 4:** Correlation of patient characteristics in neoadjuvant treatment and the prevalence of palliative treatment.

Characteristics	Odds Ratio (95% CI)^1^	*p*-value
Patient Race / Ethnicity		
No information	–	
White	1.000 (0,958; 1,045)	0,989
Black	0.979 (0,907; 1,057)	0,589
Mixed races	1.015 (0,970; 1,062)	0,523
Asian	0.898 (0,796; 1,013)	0,080
Indigenous	2.915 (0,995; 9,582)	0,058
Patient Age at Treatment Start (Prior Chemotherapy)	0.992 (0,990; 0,993)	**< 0**,**001**
Histopathological Grade		
Grade II	–	
Indeterminate	1,046 (1,007; 1,086)	**0**,**019**
Grade I	0,897 (0,839; 0,958)	**0**,**001**
Grade III	0,975 (0,935; 1,017)	0,234
Grade IV	1,336 (1,163; 1,534)	**< 0**,**001**
Stage – AJCC		
Stage 2	–	
Stage 3	1,317 (1,256; 1,381)	**< 0**,**001**
Regional Lymph Node Involviment		
No	–	
Not Assessed	0,816 (0,779; 0,854)	**< 0**,**001**
Yes	1,089 (1,047; 1,132)	**< 0**,**001**
Radiotherapy		
No	–	
Yer	1,034 (1,000; 1,068)	0,051
Therapy anti HER2		
No	–	
Yes	0,674 (0,638; 0,712)	**< 0**,**001**
Endocrine therapy		
No	–	
Yes	0,758 (0,734; 0,783)	**< 0**,**001**
Residence Location		
Other cities	–	
Capital	0,963 (0,932-0,996)	**0**,**029**
Treatment at the place of residence		
No		
Yes	0,982 (0,952-1,012)	0,228
Region		
Northeast	–	
Midwest	1,007 (0,941; 1,078)	0,833
North	0,791 (0,721; 0,867)	**< 0**,**001**
Southeast	1,122 (1,079; 1,167)	**< 0**,**001**
South	1,005 (0,955; 1,057)	0,850

^1^OR = Odds Ratio, CI = 95% confidence Interval.

## Discussion

This was a large study conducted using open data from DATASUS, in which the intersection of provided information was performed to understand the trajectory of breast cancer patients in Brazil, enabling a longitudinal perspective based on the oncological treatments received.

The average age was 51.54 years, and 49.4% of the patients were diagnosed at or below the age of 50. In the AMAZONA trial, which included patients from stages I to III, the average age was 54 years^9^. O’Neil et al.^17^ in a study on neoadjuvant therapy in public hospitals in African countries, found similar results with an average age of 50.4 years. Compared to epidemiological studies conducted in developed countries, our study reflects a younger population, a difference attributed to the aging population in developed countries^18–20^.

In Brazil, public policy for breast cancer screening recommends biennial mammography starting at age 50^21^. Despite universal access to screening mammography in Brazil, almost 49% of the population in our study had cancer diagnosed before the current screening age, with most presenting with locally advanced disease. Robust literature indicates the benefits of initiating screening from age 40. In May 2024, the United States Preventive Services Task Force (USPSTF), along with other organizations, began recommending breast cancer screening starting at age 40^22,23^.

Approximately 85% of patients were diagnosed with locally advanced neoplasia (stage III). Challenges in accessing early diagnostic services and initiating treatment impact the initial staging and affect cure rates^24^. The prior chemotherapy protocol is primarily directed at stage III in SUS. With the new TNM prognostic staging system, patients with early-stage lesions may be considered advanced when the molecular profile is taken into account, allowing for adjustments^25^.

Around 20% of breast tumors in Brazil are HER2-positive^9^. However, our study found that only 10% of patients received neoadjuvant anti-HER2 therapy. The need for immunohistochemistry (IHC) to confirm HER2 overexpression, as well as molecular testing such as FISH in equivocal cases, represents a crucial step in the therapeutic decision-making process^6,8^. Yet, disparities in access to these diagnostic tools persist across the country. In addition to timely availability, factors such as proper sample collection, adequate tissue handling, and technical quality of the processing are essential to ensure the reliability of results. These limitations are especially pronounced in remote regions with limited healthcare infrastructure, where IHC testing is not readily available. In such areas, biological samples often need to be transported to external laboratories, sometimes located in other states, leading to prolonged turnaround times and delays in initiating targeted therapies^26^. These logistical and structural barriers likely contribute to the underutilization of anti-HER2 therapy observed in the study and underscore the broader challenges faced in ensuring equitable access to precision oncology in Brazil’s public healthcare system.

The use of neoadjuvant hormonal therapy occurred in a small sample of the study population. The benefits of neoadjuvant endocrine therapy, which has been demonstrated to be safe and effective, especially for patients who are not candidates for neoadjuvant chemotherapy, are relatively recent^27,28^. Currently, there is insufficient robust evidence to conclude that neoadjuvant therapy significantly impacts survival in patients with Luminal A and Luminal B subtypes of breast cancer, unlike its effect on HER2-positive and triple-negative neoplasms^29^. Additionally, there is a lower percentage of hormone receptor-positive patients diagnosed at stage III, which constitutes the majority of our study population. These factors justify the lower percentage of patients receiving hormonal therapy in our sample compared to general data showing hormonal therapy use in 75% of patients^9,30^.

The correlation found between more aggressive histological grades, stage III, and the presence of compromised lymph nodes confirms poor prognostic factors observed in the latest TNM prognostic staging and other independent epidemiological studies^9,25,31^.

There is evidence that race may influence clinical outcomes, with Black race being associated with worse prognoses^18^. In the present study, the lack of statistical significance regarding race may be partially explained by several factors: the underreporting of racial data, Brazil’s intense racial miscegenation, and the fact that race is often recorded based on the subjective judgment of the attending healthcare professional. Moreover, racial disparities are closely intertwined with socioeconomic vulnerability and lower educational attainment—variables that may also have been confounded in this analysis, given that the study population was composed exclusively of patients treated within the public healthcare system^32,33^, which is predominantly accessed by Black and mixed-race individuals from low-income backgrounds.

Regarding the geographic distribution by region, the Southeast region is a major population hub in Brazil, serving as the primary destination for migration from rural areas and exhibiting higher rates of urbanization^6^. It has a higher life expectancy and a greater incidence of breast cancer, with an average rate of 52.3 cases per 100,000 inhabitants, which stands out compared to other regions, particularly the North, which has an adjusted rate of 27 cases per 100,000 inhabitants^1^. However, when evaluating mortality, a Brazilian study revealed a mortality rate 20 times higher for breast cancer patients in the North compared to urban centers in the Southeast^34^.

Access to healthcare services is limited, and patients often do not receive assistance in severe cases, such as the need for palliative treatment, increasing the number of deaths without proper care^33^. This could explain the regional distribution of neoadjuvant treatments and the higher prevalence of palliative treatments in the Southeast region.

The palliative treatment rate in 5 years found in our study was 36%. Shafaee et al. observed a 5-year recurrence rate of 23% in Texas patients and 21% in Brazilian hospitals, though with around 40% and 31% of patients diagnosed at stage I, respectively^12^. In the meta-analysis conducted by Pathak et al. comparing neoadjuvant and adjuvant therapy, the average prevalence of metastatic disease was 26.8%^35^. Outcomes observed in real-world settings are often less favorable than those reported in randomized clinical trials, largely due to the complexity of routine clinical practice, which involves more heterogeneous patient groups, inconsistencies in treatment implementation, and limitations imposed by institutional capacities, operational workflows, and individual patient circumstances^8^.

When observing the prevalence rate of palliative treatment by type of neoadjuvant therapy performed, patients who received anti-HER2 therapy, with or without endocrine therapy, had a lower rate of palliative treatment compared to patients who only received chemotherapy. Ensuring early immunohistochemistry and access to anti-HER2 therapy directly impacts the reduction of recurrence and mortality^36,37^. In our study, it is not possible to measure the percentage of patients who did not have access to anti-HER2 therapy, as there is no record in DATASUS regarding immunohistochemistry. However, based on previous Brazilian statistics, it is estimated that only a portion of patients with indications were correctly treated^8^.

Furthermore, for patients who are not candidates for endocrine and/or anti-HER2 therapy, the exploration of innovative strategies, such as the use of immunotherapy concomitant with neoadjuvant chemotherapy in patients with triple-negative breast cancer, may be a potential pathway for improving these outcomes^38^.

As limitations of our study, being based on secondary data, there is the inherent issue of lack of control over data quality, as well as the loss of information due to incomplete or inaccurate records. The inconsistency of information, especially from free-text fields and the lack of standardization, may lead to the loss of relevant variables. Additionally, the absence of information on mortality prevents us from assessing the rate of patients who died from breast cancer without the possibility of initiating palliative treatment.

Additionally, another potential limitation of this study is the inclusion of only stage II and III breast cancer cases, which may introduce selection bias. However, neoadjuvant therapy is not indicated for stage I disease within the treatment protocols established by the Brazilian Ministry of Health. Additionally, considering that nearly 80% of breast cancer patients in Brazil are treated within the public healthcare system, the exclusion of stage I cases is unlikely to have resulted in a significant loss of relevant information. The study, therefore, accurately reflects the real-world application of neoadjuvant therapy in the Brazilian public health context.

On the other hand, this study provides important insights into the real-world application of neoadjuvant therapy for breast cancer in the Brazilian public healthcare system. The use of a large, population-based dataset allowed for the identification of disparities in access and outcomes, contributing valuable evidence to inform strategies aimed at improving equity and effectiveness in cancer care delivery. The findings highlight the relevance of monitoring public health interventions in routine practice to guide policy and improve patient outcomes.

## Conclusion

In conclusion, this study highlights the importance of addressing disparities in access to neoadjuvant breast cancer treatments within Brazil’s public healthcare system. Despite advancements such as the incorporation of anti-HER2 therapy, significant barriers persist due to regional and socioeconomic inequalities. These challenges result in delays and inconsistencies in treatment access. Furthermore, factors such as age, histological grade, and lymph node involvement were identified as key predictors associated with the need for palliative care. The findings underscore the importance of expanding access to timely and comprehensive oncologic care, including HER2 and hormone receptor testing. Moving forward, targeted policies and strategies are essential to reduce disparities and ensure equitable, high-quality care for all patients within the public health system.

### Ethical considerations

The ethical principles outlined in Resolution 466/2012 of the National Health Council and the Declaration of Helsinki were adhered to in this study. The project was approved by the Research Ethics Committee of the Federal University of Sergipe (CEP/UFS) through registration on the Plataforma Brasil (Approval Opinion 5.544.245, CAAE 53004221.3.0000.5546). As this study involved secondary data, the use of a written informed consent form was not required.

Given that this is an observational study utilizing secondary data, the primary risk concerns the potential identification of research participants and the leakage of confidential information. To mitigate these risks, the data were handled by a limited number of researchers, ensuring confidentiality, and the anonymity of subjects was maintained in the dissemination of the results.

## Data Availability

The datasets used and/or analysed during the current study available from the corresponding author on reasonable request.
